# Fetal life malnutrition is not reflected in the relative abundance of adiponectin and leptin mRNA in adipose tissue in male mink kits at 9.5 weeks of age

**DOI:** 10.1186/1751-0147-57-S1-P4

**Published:** 2015-09-25

**Authors:** Connie Frank Matthiesen, Anne-Helene Tauson

**Affiliations:** 1 Department of Veterinary Clinical and Animal Sciences, University of Copenhagen, Copenhagen, Denmark; 2 Department of Animal Nutrition and Management, Swedish University of Agricultural Sciences, Uppsala, Sweden

## Introduction

An imbalance between fetal demand and maternal nutrient supply may lead to metabolic adaptive changes in the fetus which may benefit the fetus in the short term, by reducing fetal growth and thereby increasing nutrient availability, but might in the long term predispose the offspring to a range of diseases postnatally, such as obesity, if the changes persist.

## Methods and results

Thirty-two male mink kits born by dams fed a low (LP - 14% of ME from protein) or adequate (AP - 29% of ME from protein) protein diet for the last 16.3 ± 1.8 days of gestation were used. Kits exposed to LP supply during fetal life (FL) had significantly lower birth weight (10.3 g *vs*. 11.3 g; p=0.004) than kits provided a fetal life AP supply (FA). The dams and their offspring were fed an AP diet from parturition until weaning. At weaning, male FA and FL kits were randomly assigned to either the LP or the AP diet from 7 to 9.5 weeks of age (i.e. FA-AP, FA-LP, FL-AP, and FL-LP). The males were euthanized at 9.5 weeks of age and adipose tissues (subcutaneous, perirenal and mesenteric) were collected and analysed using q-PCR. The relative abundances of leptin and adiponectin mRNA were significantly higher in subcutaneous than in perirenal and mesenteric tissues, but not affected by fetal life protein provision.

## Conclusion

In conclusion, fetal life protein malnutrition was not reflected in adipose tissue relative abundances of leptin and adiponectin mRNA in 9.5 weeks old kits.

